# Bleachorexia—an addictive behavior to tooth bleaching: a case report

**DOI:** 10.1002/ccr3.1402

**Published:** 2018-03-25

**Authors:** Denzel Kun‐Tsung Lee, Cameron Kastl, Daniel C. N. Chan

**Affiliations:** ^1^ Division of Family Dentistry The First Dental Clinic Department of Dentistry Kaohsiung Medical University Hospital College of Dental Medicine Kaohsiung Medical University Kaohsiung Taiwan; ^2^ Department of Restorative Dentistry Health Sciences Center Seattle Washington

**Keywords:** Addiction, bleaching, carbamide peroxide, hydrogen peroxide, whitening

## Abstract

Bleachorexia, addiction to tooth bleaching, is a behavioral disorder similar to anorexia. The patient feels that their teeth are always not white enough and continues to use whiteners to obtain a “perfect” smile. Such behavior falls under the category of a body dysmorphic disorder and may need medical counseling.

## Introduction

Bleachorexia is defined as an unhealthy obsession with whitening one's teeth. This case report presented a 55‐year‐old female patient who suffered from sloughing and spontaneously bleeding gingiva over mandibular left premolar area after continuously using mouthwash and at‐home bleaching products twice a day for months. After careful history‐taking and evaluation, the patient was advised to discontinue her routine. At the two‐week recall appointment, all clinical signs and symptoms had subsided. The patient was counseled, monitored, and followed up for 6 months. This study describes the possible adverse combination effects of bleaching products and mouthwash. A brief protocol is suggested for managing bleachorexic patients.

At‐home bleaching was introduced in 1989 1. The low cost, effective outcome, and convenience of self‐application made at‐home bleaching a more popular procedure than in‐office technique 2. Such popularity stimulated the flourishing of over‐the‐counter (OTC) products and techniques. OTC bleaching products are available in drug stores, supermarkets, mall kiosks, and on the Internet as an alternative to bleach without dentist supervision. These OTC products include rinses, toothpaste and brushes, strips, paint‐on gel, floss, and tray‐based tooth whiteners. Many such products have no scientific studies to back them up and often are ineffective. Unfortunately, these products fall into the gray zone with no government regulation. Legislation varies widely in different countries regarding OTC dental bleaching 3.

In the past two decades, with the raising of self‐awareness of dental esthetics and promotions by electronic and print media, many individuals engage in improving their appearance through self‐diagnosis and “whiten” their teeth with OTC products 4, 5. People who misuse and abuse these bleaching products are in a condition called “bleachorexia” or “whitening junky,” somewhat similar to body dysmorphic disorder 3, 4. These “bleachorexic” abuse bleaching techniques and products cause teeth erosion, extreme sensitivity, and gingiva irritations 6.

## Clinical Case Report

A 55‐year‐old female patient presented to our dental clinic for routine dental hygiene cleaning. Her chief complaint was sensitivity around her mandibular gums. Her dental history included regular teeth cleanings, amalgam/composite resin restorations, and crown fabrications. There were no existing cavities that can become sensitive because of her habits.

She reported her oral home care included daily brushings and flossing after eating. Before bedtime, she used the mouthwash (Listerine^®^; Johnson & Johnson, NJ) after brushing as long as possible until it began to irritate her gums. She also mentioned that she used at‐home bleaching products (5 min Bleach Whitening Gel; Plus White, CCA Industries Inc., NJ) twice a day at least a few times per week.

The patient's salient medical history included chronic obstructive pulmonary disease (COPD), asthma, fibromyalgia, and irritable bowel syndrome. On top of the OTC products, she is currently taking several prescribed medications to take care of her medical conditions. They are listed in Table 1. The prescribed medications were screened for oral manifestations. None was found to be a possible culprit for her symptoms. She was also allergic to nickel. Her health history was negative for alcohol and drug, but she used cigarettes for 35 years. She had no specific dental complaint other than mentioning that she felt sensitivity on her gums when she brushed her mandibular posterior teeth.

**Table 1:** Current medications that might affect oral mucosa

Current medications	Active ingredients	Action	Oral manifestations
Prescribed medications			
Estradol	Estradiol	Treat symptoms of menopause Prevention of osteoporosis in postmenopausal women Replacement of estrogen in women with ovarian failure	NA
Medroxyprogesterone	Medroxyprogesterone acetate	Regulate ovulation and menstrual periods. Decrease the risk of endometrial hyperplasia while taking estrogens. Prevent overgrowth in the lining of the uterus in postmenopausal women who are receiving estrogen hormone replacement therapy.	NA
Relpax	Eletriptan hydrobromide	Treat migraine headaches	NA
Naproxen	Naproxen sodium	Pain reliever and fever reducer	NA
Qvar	Beclomethasone dipropionate	Prevent and control asthma symptoms	NA
Proair	Albuterol sulfate	Treat or prevent bronchospasm	NA
Metoclopramide.	Metoclopramide hydrochloride	Increases muscle contractions in the upper digestive tract.	Uncontrolled tongue movement
OTC medications			
Plus White	Hydrogen peroxide (6–10%)	Teeth whitening	Nonspecific ulceration and mucositis
Listerine	Alcohol (21.6%); Eucalyptol (0.092%), Menthol (0.042%), Thymol (0.064%), Methyl salicylate (0.60%)	Antiseptic	Irritants; can produce a burning or painful sensation on ulcerative mucosal surfaces.

**Figure 1:**
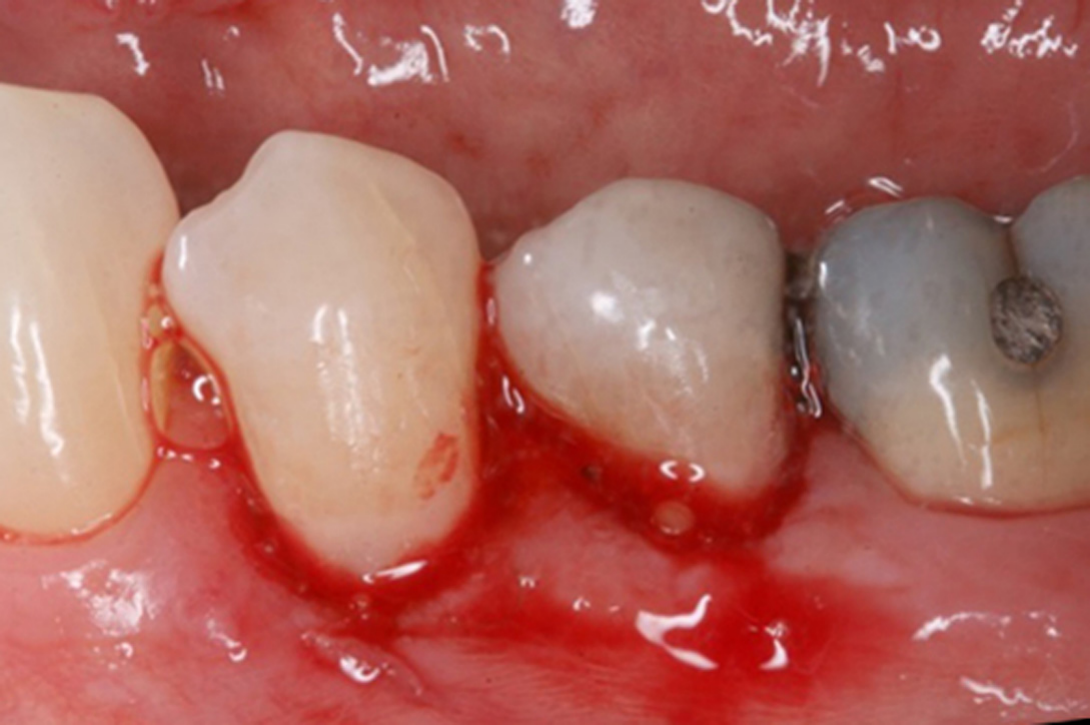
Tooth # 20 and 21. Buccal gingiva showing sloughing and spontaneous bleeding on touching.

**Figure 2:**
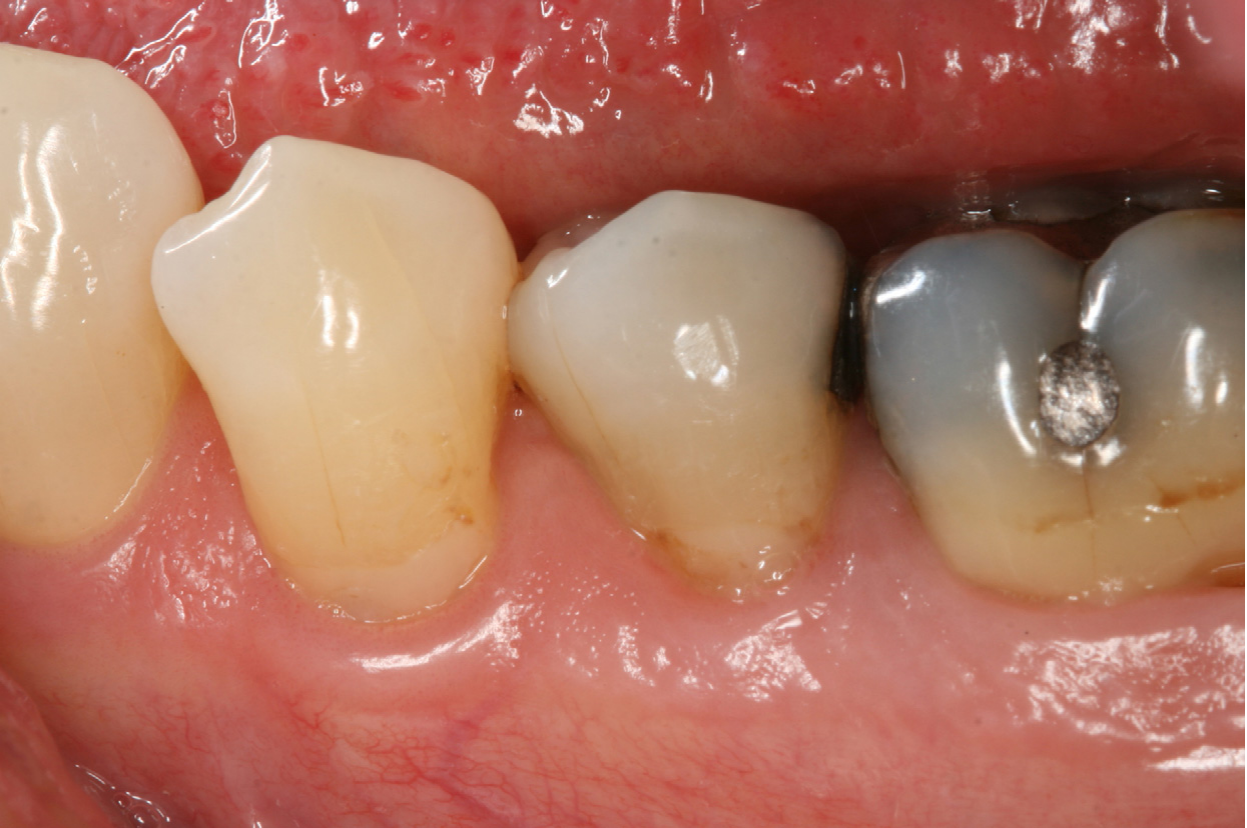
Tooth #20 and #21. Follow‐up after 6 months.

The intraoral examination revealed relatively healthy gingiva except the mandibular gum area. In addition, clinical examination revealed generalized abrasion, mild plaque and calculus over mandibular anterior teeth, and non‐carious cervical lesion on tooth #20, 21, and 28. Directly visible on the patient's oral mucosa over these areas were generalized sloughing of epithelium and spontaneous bleeding upon touching (Fig. 1). After discussing, the patient was aware of the sloughing mucosa over her sensitivity areas. She was also told that overuse of both the mouthwash and the bleaching kits could be the cause of the chemical irritation to the oral epithelium and her mucosa sloughing. She was advised to stop using the bleaching products as well as to hold the mouthwash for only 15–30 sec at a time. After 2 weeks and 6 months, the patient came back for the follow‐up and no mucosal sloughing and no more sensitivity was noted (Fig. 2).

## Discussion

The patient presented herself well and appeared to be articulate, intelligent and was very conscious of her appearance. Her motive for excessive OTC products usage was prompted by her 35‐year smoking habits. Her COPD is probably caused by smoking. Even though the patient was educated and aware of her gingival conditions, it never occurred to her that the condition could be caused by her habits. Similar abusive use of these self‐medication agents has been reported especially in young patients 7. This further underlines the importance of proper treatment planning and dentist‐supervised bleaching procedures.

In the evaluation of multifactorial causes of a clinical scenario, it is often not possible to pinpoint the exact reason. The patient claimed that she brushed her teeth until it hurts. It is true that over‐brushing can lead to sensitive teeth and receding gums, but not to the extent of spontaneous sloughing of soft tissue and bleeding. From the clinical examination, we did not notice any intrinsic and extrinsic stains. Yet the patient used a relatively strong OTC bleaching agent, 6–10% hydrogen peroxide, twice a day for several times a week. We believe that her gingival symptoms are attributed more to the compulsive bleaching action, compounded by her insistent brushing and use of mouthwash.

### Potential risk associated with mouthwashes and bleaching agents

Our patient combined the mouthwash and at‐home bleaching products to improve the appearance of her teeth even though her teeth were without any intrinsic and extrinsic stains. Her continued quest resulted in her teeth sensitivity and gingiva sloughing and bleeding.

The types of bleaching agents and the mechanisms for bleaching have been thoroughly reviewed in the literature 8, 9. Of interest to our evaluation is the concentration of the active ingredient. Compared to the concentration used for in‐office bleaching range from 25% to 40%, at‐home bleaching only contains 3–9% hydrogen peroxide. Haywood et al. have shown that lower concentrations minimize sensitivity. Unfortunately, for the purpose of efficacy, current at‐home bleaching products are trending to elevate the hydrogen peroxide concentration up to 15% 10. The increased concentration may have contributed to increased side effects. We have discovered that at‐home bleaching brand that she used contain 6–10% of hydrogen peroxide depending on the type. Surprisingly, the concentration was not prominently listed on the product ingredient panel. We had to go to some length on the web to discover the concentration. It must be pointed out that the regular at‐home bleaching systems usually contain 10% carbamide peroxide which is equivalent to only 3.5% hydrogen peroxide. The patient was using a relatively high concentration of the material.

### Adverse effects of mouthwash

Bhageerutty et al. 11 demonstrated that due to the short alcohol‐exposure times (consistent with daily mouth rinse use), Listerine^®^ did not alter the permeability of the oral mucosa.

Additionally, an in vitro study reported that human buccal mucosal tissue was resistant to effects on viability from ethanol and ethanol‐containing mouthwashes, with no adverse effects 12.

The patient reported that she was brushing as long as possible with the mouthwash until it began to irritate her gums. It would be prudent to advise the patient to use the product according to manufacturer's instructions.

### Adverse effects of bleaching agents

Safety issues and adverse effects of tooth bleaching systems are well documented due to the toxicity of hydrogen peroxide. Post‐treatment tooth hypersensitivity, gingival/oral mucosa irritations, alternations of tooth enamel surfaces, and changes in restorative materials are commonly reported. The degree of these adverse effects depends on the level of hydrogen peroxide concentration, duration, and methods of applied bleaching procedures 13. A study by Bruzell and colleagues reported at‐home bleaching was correlated with 50.3% of tooth sensitivity cases and 14% of gingival irritations 14. Although the adverse effects of tooth sensitivity and gingival irritations decreased due to adding desensitizing agents into bleaching products 15, the incidence of gingival irritations ranged from 0% to 62% depending on application methods: strips, gel in tray, paint‐on gel, or films 16. Additionally, Kristen and others also pointed out the use of a reservoir in the customer tray for at‐home bleaching resulted in higher rates and higher intensities of gingival inflammation 17. In addition, OTC system does not have a custom fitted tray and is loose like the reservoir design tray. Such system is prone to result in higher gingival inflammation.

Bleaching efficacy can be influenced by a variety of factors: gender and age of patients, initial tooth color, type and concentration of peroxide compounds, application procedures (contact time and frequency), and all these factors also affect the subsequent stability of accomplishments in bleaching. Among these various factors, the contact time of bleaching materials to tooth enamel surface is the most influential factor. In this case, patient obviously abuses the contact time using it too often, as much as twice a day for several days according to the patient. However, the effect of hydrogen peroxide in combination with alcohol or mouthwash has not been studied and is unknown. The combination of the vigorous brushing, mouthwash, and hydrogen peroxide may be a problem especially if the tissue is traumatized by the aggressive brushing habits of the patient.

### Bleachorexia and its management

The availability of OTC bleaching products provides an easy opportunity for people to bleach their teeth. Those who are in a never‐ending quest for a brighter smile and white teeth overuse in‐office and at‐home bleaching products resulting in a condition described as “bleachorexia.” This new term, a blend of bleach and ‐orexia (meaning appetite), was first coined by Dr. Jablow in 2005. The condition was also featured by American Dental Association 6.

For immediate action in the presence of adverse effects, we should recommend patient to stop usage of all bleaching products. Monitor the patient weekly to see whether their condition improves. Dentists should inform patients with bleachorexia that long‐term bleaching procedure has multiple oral health risks.

For longer term counseling, we should help the patient set more realistic expectations about tooth bleaching products. We should inform the patient that the teeth reach a maximum lightening, after which no further treatment of any type will increase. Also, when they stop bleaching, there is a slight relapse of about 1/2 shade, but when that stops, they are stable for years. Dentist should also emphasize that discoloration of teeth comes with the normal physiological aging process. Patients should be advised to avoid as much as possible the factors that causes stains and discoloration of teeth, such as coffee, tea, and red wine.

Patients with bleachorexia obsession behave like patients with body dysmorphic disorder. Some recent studies also pointed out that a preoccupation with physical appearance was a motivating factor for undergoing certain types of cosmetic dental procedures (including teeth whitening) 18, 19. Treatment for body dysmorphic disorder primarily includes psychotherapy in the form of cognitive behavioral therapy. In our case, counseling and constant monitoring was successful in stopping the patient from the abuse. Patients with severe bleachorexia obsession should be referred to a primary physician.

Medications have been used successfully to treat body dysmorphic disorder are typically antidepressant medications, including citalopram (Celexa), escitalopram (Lexapro), fluoxetine (Prozac), fluvoxamine (Luvox), fluvoxamine CR (Luvox CR), paroxetine (Paxil), paroxetine CR (Paxil CR), and sertraline (Zoloft). Prescribing these medications is outside the scope of practice of dentistry; therefore, severe cases should be referred.

## Conclusion

Our case report supports the ADA recommendation that if one chooses to use a bleaching product, one should only do so after consultation with a dentist. Dentists should also be acquainted with OTC products to be able to inform their patients. When a bleachorexic patient is encountered, behavioral modification should be our first goal. Severe cases should be handled like patients with body dysmorphic disorder and referred to a primary physician for cognitive behavioral therapy and medications.

## Disclaimer

The clinical pictures were digitally cropped and orientated for comparison.

## Authorship

K‐TDL: contributed to conception and design, contributed to acquisition, and drafted manuscript. CK: contributed to acquisition and drafted manuscript. DCNC: critically revised manuscript, gave final approval, and agreed to be accountable for all aspects of work ensuring integrity and accuracy.

## Conflict of Interest

None declared.
